# Assessing the impact of anti-microbial showerheads on the prevalence and abundance of opportunistic pathogens in shower water and shower water-associated aerosols

**DOI:** 10.3389/frmbi.2023.1292571

**Published:** 2023-11-02

**Authors:** Sarah Pitell, Sarah-Jane Haig

**Affiliations:** ^1^ Department of Civil and Environmental Engineering, University of Pittsburgh, Pittsburgh, PA, United States; ^2^ School of Public Health, University of Pittsburgh, Pittsburgh, PA, United States

**Keywords:** opportunistic pathogen, DWPI, Legionella, NTM, 16S rRNA, antimicrobial showerheads, bioaerosolization

## Abstract

Respiratory infections from drinking water-associated pathogens that can cause infections in the immunocompromised (DWPIs) are increasing, yet knowledge of DWPI aerosolization and if dynamics are DWPI-specific is lacking. Although there are several DWPI mitigation strategies, the use of antimicrobial showerheads is one of the easiest and most economical. There are many manufacturers and designs of antimicrobial showerheads that claim to remove microorganisms from shower water, yet all fail to assess efficacy in realistic conditions. In this study, a custom-built shower laboratory housing triplicates of three different showerheads (antimicrobial filter-based, antimicrobial silver-embedded and conventional acrylonitrile butadiene styrene plastic) were used to assess the physiochemical and microbial dynamics in shower water and respirable shower water-associated aerosols (1µm – 5 µm) over the course of 84 days. Collectively, findings from the study suggest that showerheads marketed as antimicrobial produce similar chemical and DWPI water quality to non-antimicrobial showerheads (p= >0.05) when operated under real-world conditions, however marked differences in the rare microbial community were present. In addition, although there were no differences in absolute DWPI abundance between showerhead type, each DWPI peaked in concentration at a different biofilm ages, suggesting that potential DWPI inhalation risk is DWPI- specific and influenced by the number of days of operation of the showerhead.

## 1 Introduction

Respiratory infections from drinking water-associated pathogens that can cause infections in the immunocompromised (DWPIs) (C. Proctor et al., 2022) cost the US economy $2.39 billion annually (Collier et al., 2021). Today, the incidence of waterborne disease outbreaks in the United States attributed to DWPIs not regulated by the US EPA (e.g., *Legionella pneumophila*, nontuberculous mycobacteria (NTM), *Pseudomonas aeruginosa*) appear to be increasing (Feazel et al., 2009; Cunha et al., 2016; Adjemian et al., 2017; Benedict, 2017), and far exceeds disease incidence caused by regulated fecal-borne pathogens (Feazel et al., 2009; Cunha et al., 2016; Adjemian et al., 2017; Benedict, 2017). While present naturally in the environment, DWPIs are typically not quantified in finished drinking water (DW) leaving the treatment plant, but can multiply within the distribution system and in building plumbing, often within biofilms (Berry et al., 2006). Surfaces in building plumbing favor the formation of these biofilms, allowing DWPIs to persist and grow (Angenent et al., 2005; Feazel et al., 2009; Craun et al., 2010; Moritz et al., 2010), likely explaining the higher abundance of DWPIs in water at the point of use *(* Haig et al., 2020) and connection to several clinical infections (Bornstein et al., 1986; Falkinham, 2011). Previous work has been conducted to evaluate initial colonization in virgin plumbing materials, and has found that the microbial composition of DW biofilms change over time, with marked differences occurring between the installation (day 0) and 13 days, 14-42 days after installation, and >43 days after installation (Morvay et al., 2011). These changes in biofilm composition within the pipe may influence the DW microbiome exiting fixtures due to sloughing, but little is known about these dynamics. Although human exposure to DWPIs occurs through many pathways, inhalation of DW associated aerosols has been linked to pulmonary infections (Bollin et al., 1985; Angenent et al., 2005; Falkinham et al., 2015a; Proctor et al., 2022). Despite these observations, the majority of DWPI research has focused on biogeographical surveys to identify locations where these organisms can be found and linkages with physiochemical parameters, but critical steps in the DWPI transmission pathway—aerosolization and mitigation approaches remain poorly understood.

Given that the average adult showers for 8 minutes every day (Wilkes et al., 2005) and that building plumbing has consistently detectable DWPIs (Falkinham et al., 2015b; Haig et al., 2020), the most promising DWPI mitigation location would be at the final point of use (e.g., showerheads). Currently, to help reduce the risk posed by DWPIs in household and healthcare shower water, individuals and facility managers use a variety of approaches spanning from large-scale engineered solutions [periodic thermal (Stout et al., 1998; Bédard et al., 2016) or chlorine shocking of building plumbing (Orsi et al., 2014)] to more economic and tunable approaches such as the use of antimicrobial showerheads. The designs of antimicrobial showerheads fall into one of two categories: chemically mediated antimicrobial activity (e.g., use of silver and or copper) or physiochemical antimicrobial activity [e.g., filtration through a media bed or block (Velten et al., 2011)]. Regardless of the type of antimicrobial showerhead, all claim to reduce or eliminate microorganisms from shower water. Such claims are substantiated by culture-dependent assessment approaches used by regulators to detect pathogens, even though it is widely known that these approaches can provide false-negative results if DWPIs exist in the viable but non-culturable (VBNC) (Ramírez-Castillo et al., 2015) state, or if they are present below the method detection limit. Furthermore, manufacturers follow ISO 22196:2011 (In2itive, 2011) to test their antimicrobial material, which does not simulate the shower environment and focusses on quantifying microbial reduction of non DWPI organisms (e.g., *Escherichia coli*) using culture-based methods, and completely overlooks the exposure route – aerosols (The Efficacy of the Medi-Shower Silver Impregnated Showerhead, 2017; Yetiş et al., 2022a; Yetiş et al., 2022b).

Previous studies have assessed DWPI concentration in aerosols produced from showers, however, these studies although pioneering have many drawbacks; namely the microbial assessment in all aerosol size fractions (Bollin et al., 1985; Thomson et al., 2013) (including fractions which cannot be respired) (Shen et al., 2022), unrealistic shower operation and sampling approach (Shen et al., 2022), the use of hard impaction for collection (Bollin et al., 1985; Thomson et al., 2013), which reduces recovery and can distort aerosol size, and the lack of replication. Given these shortcomings there is an immediate need to quantitively assess the efficacy of antimicrobial showerheads on both their produced water and respirable shower water-associated aerosols (1µm – 5 µm) under realistic conditions.

This study compared water quality, DWPI abundance and microbial community composition in shower water and respirable shower water-associated aerosols between antimicrobial and conventionally used acrylonitrile butadiene styrene (ABS) plastic showerheads using a custom full-scale shower laboratory to simulate real world showering conditions. Two antimicrobial showerheads were used in this study: one marketed to contain silver impregnated into the plastic, and the other contained a proprietary multi-stage filter. Shower water and their associated aerosols were collected from triplicates of each showerhead biweekly over the course of 14 weeks (84 days). All samples were analyzed for DWPI abundance using droplet digital PCR, microbial community dynamics using 16S rRNA amplicon sequencing, and a variety of physiochemical parameters.

## 2 Materials and methods

### 2.1 INHALE shower laboratory set up and tested showerheads

The INHALE shower laboratory at the University of Pittsburgh, PA, consists of three full-scale shower stalls (Sterling Ensemble 34 in. x 42 in. x 77 in) connected to their own separate 50-gallon electric water heater (Bradford White Corporation, Model Number: RE350S6 – 1NCWW), using municipal water supplied by the City of Pittsburgh after transit through building plumbing. The water pressure feeding into the laboratory is 60 psi, with a 56 psi pressure measured at the outlet. In each stall, there are three showerheads that are controlled with independent valves, allowing for triplicate studies to be run (nine showerheads overall) (**Supplementary Figure S1**). The shower laboratory was constructed with virgin copper piping for the plumbing, and contains a thermomixing valve on the outlet of each water heater that is set so that the water coming out of the showerheads is 40°C, the average shower temperature of Americans (Wilkes et al., 2005). Each showerhead tested had an output flow of 2.5 gpm. A ¾” hole was drilled in each Plexiglas shower stall door 154 cm from the shower floor in order to collect bioaerosols present in the average American adult’s respirable zone (McDowell et al., 2008). Each showerhead was flushed daily for 8 minutes to simulate an average American’s shower (Wilkes et al., 2005) and to replicate real-world shower impacts on the biofilms within the pipes.

Three different types of showerheads were installed in triplicate in the INHALE shower laboratory: a commonly used and widely available showerhead made from acrylonitrile butadiene styrene plastic (ABS) and two marketed antimicrobial showerheads (one containing silver nanoparticle technology embedded in the plastic polymer; referred to from here forward as silver embedded, and the other showerhead was an ABS plastic showerhead that contained an in-line proprietary filter containing zinc, calcium, and copper; referred to from here forward as filter-based). The silver-embedded showerhead was marketed to be bacteriostatic and to prevent the formation of biofilm inside the showerhead and hose. The filter-based showerhead is marketed to remove bacteria from the resulting shower water in addition to removing iron and chlorine. All tested showerheads were obtained directly from their respective manufacturers. Prior to the installation of the showerheads to be assessed, ABS heads were installed on each of the three outlets in each stall and flushed daily for 8-minutes for 1 month to eliminate water quality artifacts due to stagnation.

### 2.2 Shower water and shower water associated bioaerosol collection

Water and respirable aerosols (<10 µm in diameter) samples were collected in tandem biweekly over the course of 3 months (yielding 7 sampling events in total) from each showerhead. This time frame was chosen following the manufacturer’s guidelines to replace the silver-embedded showerhead after 60 days of use: the sampling period of 84 days allowed for studying its performance during its marketed effective treatment period, and past its recommended timeframe. The aerosol sampler was turned on at the same time as the shower, and an 8-minute composite water samples were taken at the same time as aerosol collection. The shower and aerosol sampler continued to run for a total of 20 minutes after the water was collected, when the shower and aerosol sampler was turned off. To prevent aerosol contamination from one showerhead to the others and to allow aerosol abundance to return to baseline, one showerhead at a time was sampled with a gap of at least 1 h between heads.

Briefly, water sampling entailed collecting a 1.5 L composite sample taken over 8 minutes after the shower water reached temperature for each head into a sterile Nalgene bottle. Allowing the water to reach showering temperature before sampling was to simulate what a person showering would come into contact with. 1 L was immediately filtered through a 0.2 µm polycarbonate filter (Millipore, Cork, Ireland) and the filter was stored at -20°C prior to extraction, while the remaining water was used for water chemistry analysis. Deionized water was processed identically to the shower water samples as a negative control.

Bioaerosols were sampled using the 110A Spot Sampler by Aerosol Devices Inc. (Aerosol Devices, Fort Collins, CO), with the addition of a SCC1.829 cyclone (Mesa Labs, Lakewood, CO) that allowed for collection of respirable aerosols (<10 µm in diameter). Following the approach of Nieto-Caballero et al., 2019 bioaerosols were collected into 0.6 mL of RNAlater (Thermofisher, Waltham, MA) using antistatic tubing for 20 minutes to ensure sufficient biomass was collected. Samples were stored at -20°C prior to extraction. Background samples were taken prior to each sampling event where aerosols were collected in the INHALE shower laboratory for 20 minutes with no showers running, and aerosol control samples were taken at sampling events 1, 4, and 7 by installing a HEPA filter in-line with the sample tubing.

### 2.3 Water quality measurements

Twenty water quality parameters (**Supplementary Table S1**) were measured using previously described methods(Haig et al., 2020). Ammonia, orthophosphate, free chlorine, and total chlorine concentrations were determined at the time of collection using a portable DR900 spectrophotometer (Hach, Loveland, CO, USA). Temperature and pH were monitored onsite using a portable pH and temperature meter (HANNA Instruments, Woonsocket, RI). Total and dissolved organic carbon were measured using the Shimadzu TOC-L analyzer using the subtractive method (Shimadzu, Kyoto, Japan). Total and dissolved iron, lead, copper, silver, calcium and magnesium were determined using inductively coupled plasma mass spectrometry (PerkinElmer NexION 300 ICP-MS, PerkinElmer, Waltham, MA). Prior to analysis, all dissolved organic carbon and dissolved metal samples were prepared by passing water through a 0.45 µm nylon syringe filter (Thermofisher, Waltham, MA) primed with 5 mL of sample. Deionized water was processed in the same way to samples as controls. All analyses, except pH, and temperature, were performed in triplicate and the coefficient of variation was at most 13%.

### 2.4 DWPI quantification

DNA from collected water and aerosol samples were extracted using the Fast Spin DNA Extraction kit (MPBio, Irvine, CA), where the extracted DNA was eluted into 100 uL of DES (Haig et al., 2018) and stored at -20°C until further analysis. Extraction controls were performed for each extraction kit where nothing was added to the extraction kit reagents, and filter controls were processed by extracting filters that had no material passed through it for each filter manufacturing batch. Absolute densities of total bacteria, *Legionella pneumophila, Pseudomonas aeruginosa*, and NTM were determined using droplet digital polymerase chain reaction (ddPCR) (QX200, Bio-Rad, Hercules, CA) targeting the 16S rRNA gene and taxon specific genes, respectively (**Supplementary Table S2**). All samples were analyzed in duplicate along with negative controls (field blanks, extraction blanks, and ddPCR blanks of molecular grade water as the template) and gblock positive controls of each amplicon (Integrated DNA Technologies, Inc., Coralville, IA). Each 22 µL ddPCR reaction contained 11 µL of EvaGREEN supermix (Bio-Rad, Hercules, CA), 0.625 mg/mL bovine serum albumin (Invitrogen Corporation, Waltham, MA, USA), 0.2 uM primers (Integrated DNA Technologies, Inc., Coralville, IA) (**Supplementary Table S2**), 7.57 µL of water, and 2 uL of the extracted template DNA at an assay specific dilution. Droplets were generated to a 20-μL reaction volume using the automated droplet generation oil for EvaGREEN (Bio-Rad, Hercules, CA), and the plate was heat sealed. PCR was performed using a C1000 Touch thermal cycler (Bio-Rad Laboratories) within 15 min of droplet generation using the reaction conditions presented in **Supplementary Table S2** Within 1 h of PCR completion plates were ran on the droplet reader for quantification. Thresholds were set for each ddPCR assay (Supplementary Procedure S1) and the absolute density of the target taxa were determined using Quantasoft v1.0.596 following the method described by Lievens et al. (Lievens et al., 2016). Paired water and aerosol samples were compared after DWPI quantification to assess DWPI partitioning by calculating the ratio of DWPIs in the aerosol phase and water phase.

### 2.5 16S rRNA gene amplicon sequencing

16S rRNA gene amplicon library preparation and sequencing were performed on water and aerosol samples at Argonne National Laboratory following the Illumina Earth Microbiome Protocol (Caporaso et al., 2012). Samples were sequenced on an Illumina HiSeq2500 with a total of 1,633,966 raw reads generated. Microbiome analysis was performed using QIIME2 (version 2020.2) with quality filtering performed using the method described in Bolyen et al. (Bolyen et al., 2019). Reads were assigned to operational taxonomic units (OTUs) using a 97% cutoff using the closed reference OTU-picking protocol in QIIME2 (version 2020.2) using the Silva (version 132.5) reference database. All data were processed using the University of Pittsburgh’s Center for Research Computing cluster servers.

### 2.6 Statistical analysis

All data was visualized and analyzed using R statistical software (Version 4.0.5). Significant differences (*p*-values <0.05) of parameters by head type, sample type, and over time were determined using analysis of variance (ANOVA) tests, and paired Mann Whitney U-tests. Linear mixed-effect models were developed to determine which physiochemical parameters impacted absolute abundances of DWPIs utilizing a stepwise forward and reverse approach to find the model with the lowest Akaike Information Criterion value (Haig et al., 2020). Prior to model generation, all DWPIs abundances were transformed to ensure normal distributions, all physiochemical data were scaled, and all collinear variables were assessed and removed using a variance inflation factor (VIF) values <10. Power calculations revealed no more than five explanatory variables should be included in the models. Taxonomic data generated from sequencing were Hellinger transformed prior to analysis to minimize the impact of low abundances of many taxa (Legendre and Gallagher, 2001). Pairwise dissimilarities between samples were calculated based on the Bray-Curtis dissimilarity index, and examined for temporal and spatial patterns in the bacterial community structure by Non-metric Multidimensional Scaling as implemented in the Vegan package in R (Oksanen et al., 2022). Significant differences in the microbial community compositions (Shannon diversity index, Chao’s richness, and Pielou’s evenness) based on showerhead age and sample type were determined by ANOVA. Relationships between environmental parameters and patterns in microbial community composition were examined by redundancy analysis (RDA) with significance tested by ANOVA after reducing the overall suite of environmental variables with VIF (Haig et al., 2020).

## 3 Results

### 3.1 Showerhead type did not impact shower water chemistry

Overall, there was no significant difference in effluent showerhead water chemistry between any of the head types (ABS, silver-embedded, and filter-based), despite different materials used and marketing claims (**Supplementary Table 3**). In particular, it was surprising to observe no significant differences in the concentration of chemicals expected to leach from the showerheads (i.e., organic carbon from the ABS showerhead, organic carbon and silver from the silver-embedded showerhead, and organic carbon, calcium, and copper from the filter-based showerhead) during any point of the 84-day long sampling period. In terms of additional treatment besides the antimicrobial properties of the showerheads, the filter-based showerhead claimed to remove 95% of total chlorine. However, there was no significant difference in chlorine concentration between all head types, although the filter-based heads had the lowest absolute concentration of free and total chlorine when the average values were compared (**Supplementary Table S3**).

### 3.2 DWPI presence and abundance was unaffected by showerhead type

**Figure 1:**
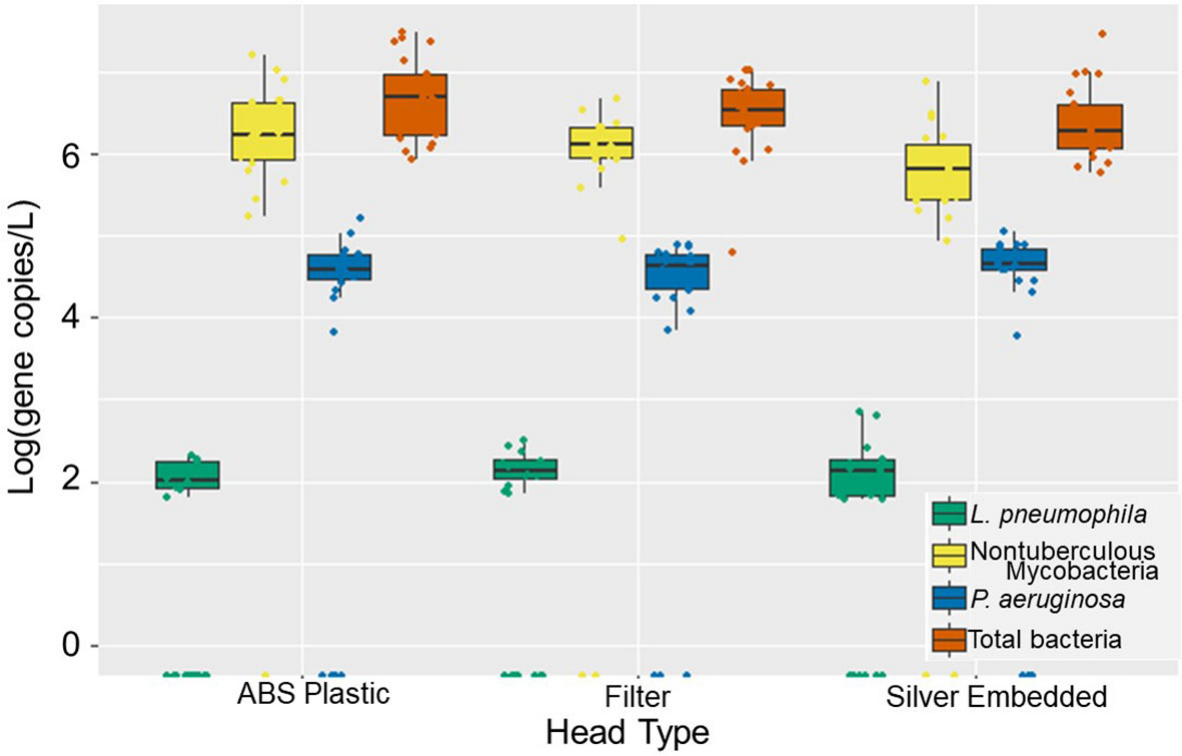
Absolute gene copy concentration of total bacteria (orange), *Legionella pneumophila* (green), *Pseudomonas aeruginosa* (yellow), and nontuberculous mycobacteria (blue) observed across 84 days of operation in ABS, filter-based antimicrobial, and silver-embedded showerheads in shower water. Each showerhead type shows all the data collected from three experimental showerhead replicates.

When the water and aerosol samples were analyzed for DWPIs and total bacteria, no statistically significant differences in the absolute abundance were found between the antimicrobial showerheads and the ABS showerheads (**Figure 1** and **Supplementary Figure S2**). Likewise there were no significant temporal changes in individual DWPI abundance between showerhead type, but there were marked differences in the behavior of each DWPI. *L. pneumophila* and *P. aeruginosa* were detected transiently in the water samples in low concentrations (88 gene copies/L and 3.9 x 10^4^ gene copies/L, respectfully), whereas NTM were consistently abundant and increased in concentration after day 42 of continuous use (**Supplementary Figure S4**). Low biomass recovered from aerosol samples likely explains why the same trends were not observed in water samples (**Supplementary Figure S3**).

Amplicon sequencing results of the whole microbial community corroborated the absolute quantification data: NMDS analysis revealed no significant clustering in samples based on showerhead type (**Supplementary Figure S5**), and RDA analysis confirmed that showerhead type was not a significant parameter in explaining differences in microbial community.

Simple linear models were generated using the absolute quantification data and physiochemical data to determine which parameters influenced DWPI abundance in water, aerosols, and partitioning (the fraction of DWPIs measured in the aerosol phase and the DWPIs measured in the water phase) (**Table 1**). Models generated from the aerosol and partitioning data explained lower levels of variance or couldn’t be generated due to the number of samples under the limit of quantification for a specific target. The models identified different parameters for each target DWPI and total bacteria for each phase investigated that contributed to explaining variance, however there were general trends that emerged in these models. In the models generated from the water samples, parameters commonly associated with DWPI presence and abundance were identified such as iron (Falkinham et al., 2015b) and chlorine (Kuchta et al., 1985), as well as showerhead age and type. The models from the aerosol samples identified a wider variety of parameters, along with the importance of the overall microbial load of the drinking water. Partitioning models revealed that in addition to other physiochemical parameters, partitioning of some DWPIs affected others.

**Table 1:** Summary of generated linear models. In the model components column, ± indicates positive or negative association and the percent of the variance explained by each variable is superscripted.

Model (Transformation)	Model Components		Overall Model	
			Explained (%)	*p-*value
L. pneumophila				
Water (Square root)	Showerhead Type^27%^ + Showerhead Age^2%^ + Total Chlorine^25.6%^ + Total Calcium^11.9%^ + Dissolved Copper^2.3%^		42.20	5.8 x 10^-6^
Aerosols (Square root)	Total Chlorine^21.9%^		21.90	1.1 x 10^-4^
Partitioning ratio (Logarithmic)	*L. pneumophila* in water^29%^ – NTM emission^0.5%^ – Total Chlorine^5.2%^ + Total Iron^7.1%^ – Dissolved Copper^6.4%^		49.10	3.2 x 10^-7^
P. aeruginosa				
Water (Square root)	Showerhead Age^33%^ + Free Chlorine^18%^ + Dissolved Iron^4.2%^ – Total Organic Carbon^6.6%^		61.7	1.5 x 10^-11^
Aerosols (Logarithmic)	Temperature^5.2%^ + Total Iron^14.8%^ – Dissolved Organic Carbon^5.2%^ + Total Bacteria in Water^6.8%^		32	1.4 x 10^-4^
Partitioning ratio (Logarithmic)	Total Iron^31.9%^ + Total Bacteria emission^11.2%^ + Temperature^6.4%^		49.5	8.0 x 10^-9^
Nontuberculous mycobacteria (NTM)				
Water (Square root)	Total Bacteria in water^15.9%^ – Showerhead Type^5%^ – Free Chlorine^19%^		39.9	1.3 x 10^-6^
Aerosols (Logarithmic)	pH^1.5%^ + Total Copper^13.2%^ + Total Organic Carbon^3.2%^ – Total Magnesium^9.8%^		27.8	7.3 x 10^-4^
Partitioning ratio (Logarithmic)	–			
Total Bacteria				
Water (Logarithmic)	Total Iron^23.4%^ + Temperature^10.1%^ + Dissolved Copper^4.8%^ + NTM in water^16.2%^		54.6	1.9 x 10^-9^
Aerosols (Square root)	Total Bacteria in water^24%^ – Total Iron^0.01%^ + Total Magnesium^10%^ + Dissolved Copper^3.2%^ – Total Calcium^8.6%^		45.9	9.8 x 10^-7^
Partitioning ratio (Logarithmic)	*P. aeruginosa* emission^28.7%^ + Dissolved Silver^8.8%^ – *P. aeruginosa* in water^4.1%^		41.6	5.3 x 10^-7^

Multivariate statistical analysis revealed that the most influential parameter to explain DWPI abundance was the showerhead age (days of use since installation) (**Table 1**). The importance of showerhead age is unsurprising given biofilms develop in the virgin hose and fixtures and thus begin to influence the microorganisms in the shower water and aerosols (Costerton et al., 1995). According to the manufacturers of the silver-embedded and filter-based antimicrobial showerheads, both fixtures were to inhibit biofilm formation, and thus reduce the microbial load. However, DWPI and total bacteria concentrations were comparable regardless of head type, which further supports that these antimicrobial showerheads are no more effective than conventionally used ABS showerheads under real-use conditions (**Supplementary Figures S2**–**S4**) based on molecular assessment.

### 3.3 Aerosolization behavior of DWPIs is species specific

**Figure 2:**
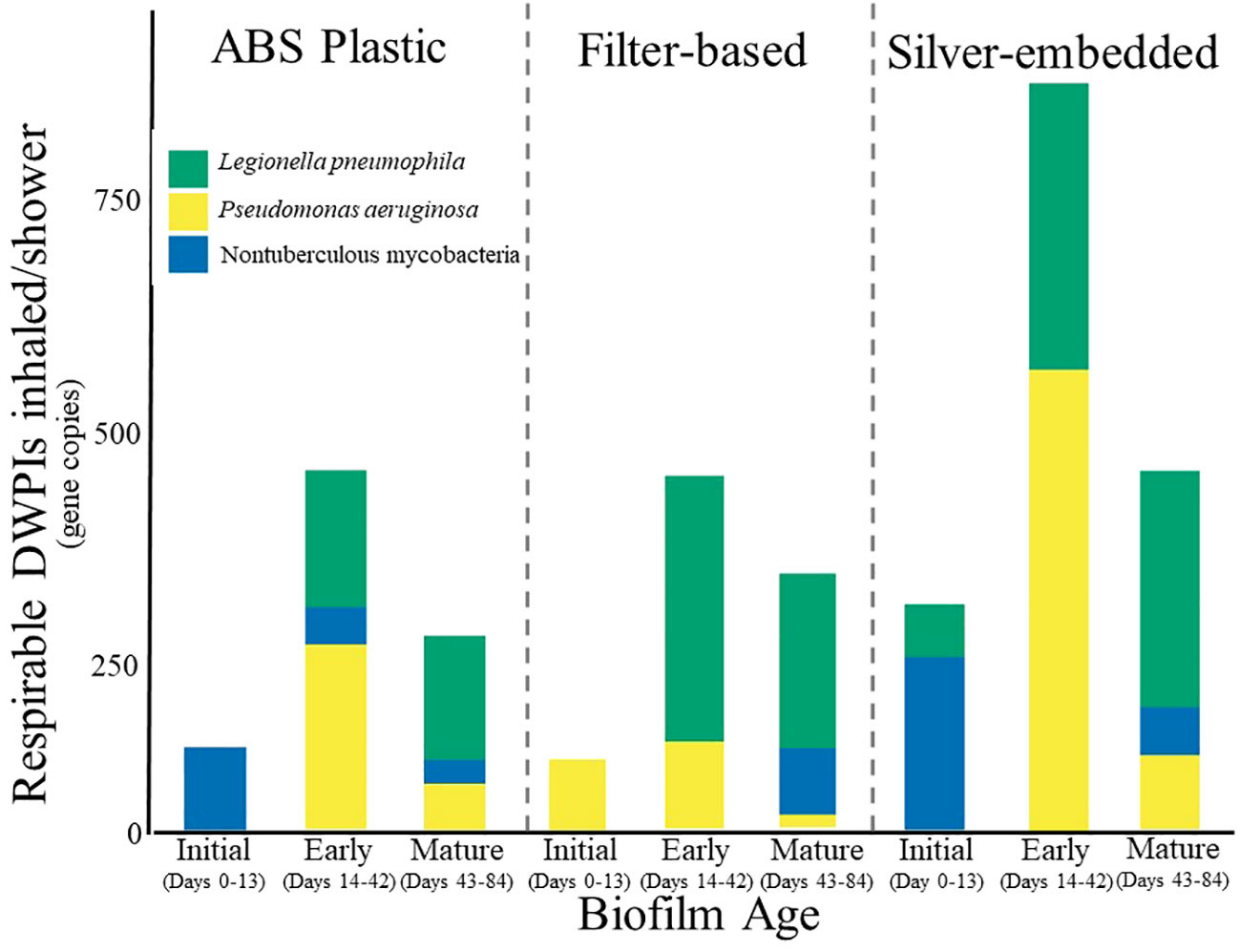
Stacked barplots of the average absolute abundance of *Legionella pneumophila* (green), *Pseudomonas aeruginosa* (yellow), and nontuberculous mycobacteria (blue) within bio-respirable shower water-associated aerosols (<10 um) produced during an 8-minute shower using ABS plastic showerhead, silver-embedded showerhead, and filter-based showerhead. Averages are based on triplicates of each showerhead type. Error bars were excluded due to the high variation in the dataset.

Despite low DWPI abundance, concentrations of aerosolized DWPIs emitted over the course of an 8-minute shower did vary by showerhead type (**Figure 2**), with each DWPI exhibiting similar peaks in concentration at the same biofilm age across showerhead types. More specifically, NTM peaked in inhalable concentration at the time of installation (0-13 days after showerhead installation) in the ABS plastic and silver-embedded showerhead samples, *P. aeruginosa* shows highest inhalable concentrations during early biofilm formation (14-42 days after showerhead installation) and *L. pneumophila* displays consistent concentrations during both early and mature biofilm age (14-42 and 43-84 days after showerhead installation, respectively). However, the ABS plastic showerhead and silver-embedded showerhead had NTM as the dominant aerosolized DWPI at the time of installation, whereas the filter-based showerhead had majority *P. aeruginosa* in the initial aerosol collection. The silver-embedded showerhead also had a greater aerosolized DWPI load during early biofilm formation (14-42 days after showerhead installation), which was almost double the abundance of the other showerhead types. The filter-based showerhead samples had low levels of NTM until the mature biofilm stage, which could either be attributed to biofilm sloughing or aerosol forming mechanisms. Collectively these results suggest that potential DWPI inhalation risk is DWPI specific and influenced by both the number of days of operation of the showerhead and the showerhead type, however it should be noted that the variability in this data obscured any statistical significance in these trends, so further work studying DWPI dynamics over time is needed.

**Figure 3:**
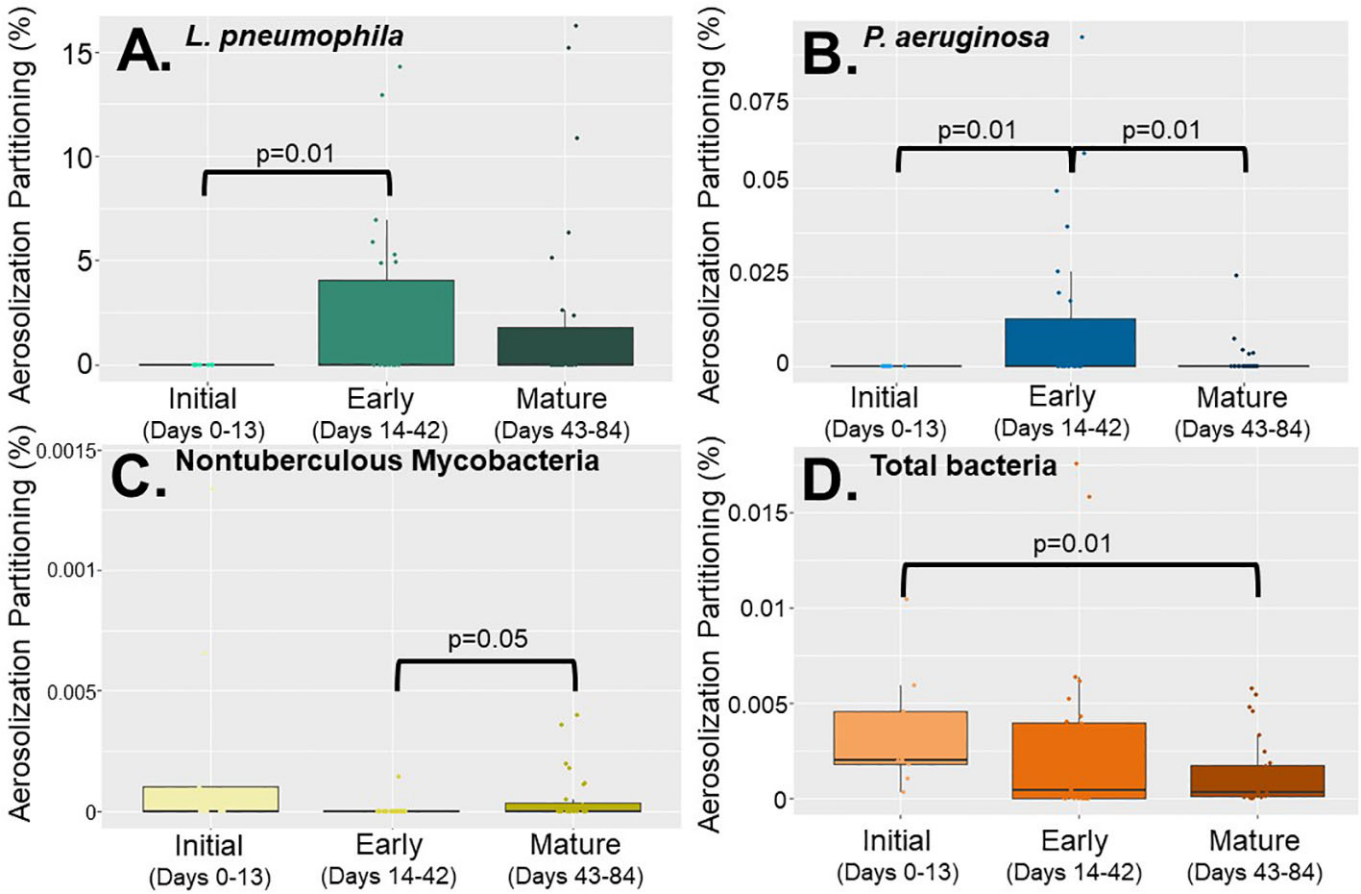
Partitioning percentages of **(A)** *Legionella pneumophila*, **(B)** *Pseudomonas aeruginosa*, **(C)** nontuberculous mycobacteria, and **(D)** total bacteria based on biofilm growth stage; initial (0 -13 days), early (14-42 days) and mature (43-84 days) using all data collected from all showerhead types.

Looking at partitioning behavior (microbial concentration in the aerosol phase divided by the microbial concentration in the water phase), no statistically significant difference in DWPI behavior was observed between the showerhead types, however silver-embedded showerheads did have higher partitioning ratios and standard deviations than the other two showerheads (**Supplementary Table 4**). Despite the lack of difference in DWPI partitioning between showerhead types, there were significant differences in individual DWPI partitioning behavior as a function of time (biofilm age) which was consistent across all showerhead types (**Figure 3**). Specifically, NTM appeared to partition at the highest frequency at time zero (**Figure 3C**) and then dissipated as the biofilm established. Whereas *L. pneumophila* and *P. aeruginosa* show their highest partitioning behavior during early biofilm formation (**Figures 3A, B**). Overall, considering both aerosolization and partitioning behavior data, antimicrobial showerheads did not significantly impact microbial aerosolization in the shower system.

### 3.4 Microbial community dynamics were phase (water and aerosol) and showerhead age dependent

**Figure 4:**
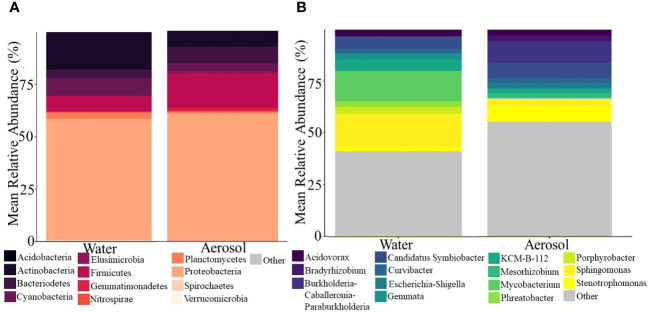
Stacked barplots showing the major **(A)** phyla and **(B)** top 10 genera in shower water and shower water-associated aerosols. Data illustrate the average abundance observed across all showerhead types and all timepoints.

RDA analysis revealed that the microbial community was significantly impacted by phase and age (days of operation), with phase explaining 7.2% and age 10.7% of the variance observed. Between water and aerosol phases, the community structure and membership of dominant taxa were surprisingly comparable despite the bioaerosolization process being known to reduce overall microbial concentrations and cause damage to cell membranes (**Figure 4**) (Shen et al., 2022). Alpha diversity analysis revealed that samples from either phase were similar in richness and diversity, but that water samples were less even than aerosol samples. Looking at the most abundant phyla (**Figure 4A**) in the water and aerosol samples, *Proteobacteria* were the dominant phyla in water (58%) and aerosol (61%) samples alongside other commonly reported DW phyla (Proctor et al., 2016; Gebert et al., 2018) such as *Firmicutes, Actinobacteria, Cyanobacteria*, and *Bacteroidetes*. However, at the more resolved genus level, there was much more variation in community composition between water and aerosol samples with only 40% of all genera being common between phases, with *Acidovorax*, *Sphingomonas*, and *Stenotrophomonas* being the most abundant. Despite many similarities in the microbiome between the aerosol and water samples, there were distinctions in the beta diversity being driven by rare taxa which explains the distinct clustering of aerosol and water samples (**Supplementary Figure S5**).

**Figure 5:**
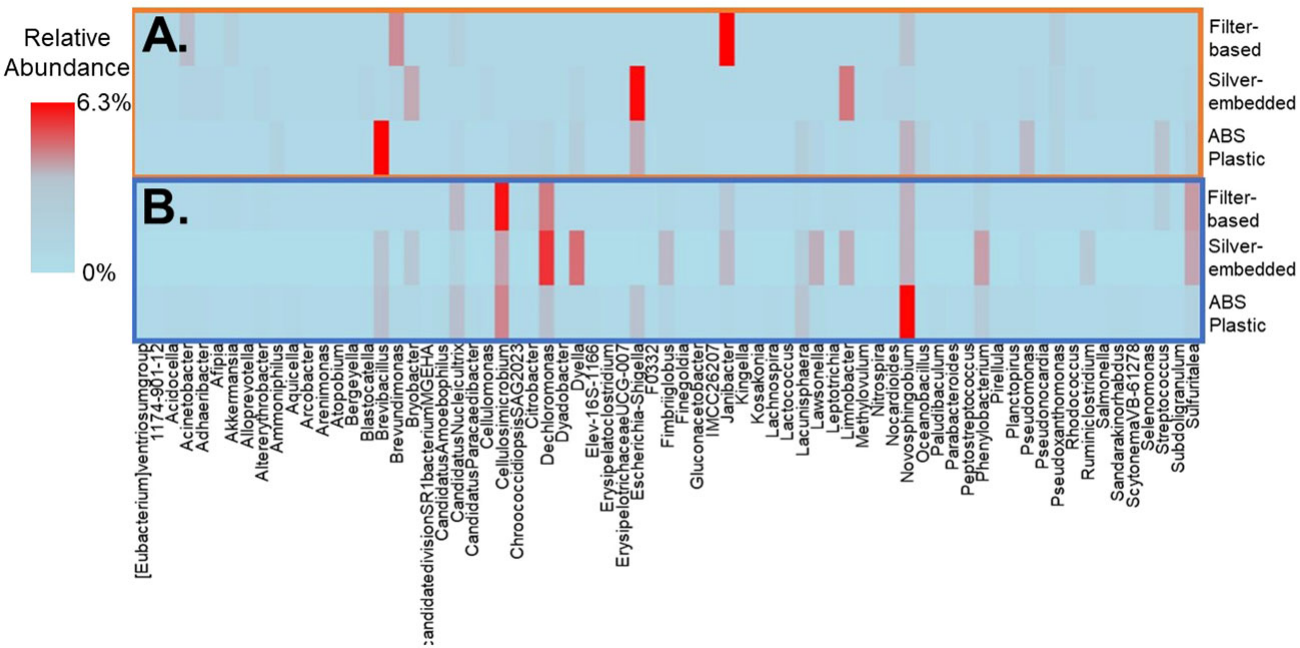
Least abundant fifteen genera membership for each sample type and showerhead type in **(A)** aerosol samples and **(B)** water samples. Data illustrate the average abundance observed across all showerhead types and all timepoints.

Collectively, across all showerhead types 27% and 21% of genera were shared in water and aerosol samples, respectively, however ABS Plastic showerheads displayed the least number of genera (99 in water and 65 in aerosols) and filter-based showerhead had the most (148 in water and 74 in aerosols). The aforementioned DWPIs were among the top ten most abundant genera shared between showerhead types and phases, as well as *Mycobacteria*. These DNA sequencing results suggest that the antimicrobial showerheads do not select for fewer taxa based on their material properties as expected, but in fact support different rare taxa compared to the commonly used ABS showerheads (**Figures 5A, B**).

**Figure 6:**
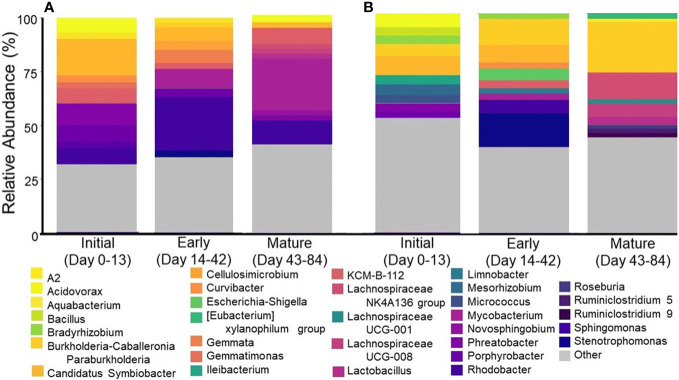
Top ten most abundant genera by biofilm establishment period for **(A)** water samples and **(B)** aerosol samples. Each bar represents an average across all showerhead types.

NMDS analysis of the water samples from this study revealed that the microbial community structure was distinct during the different stages of biofilm formation, with tight clustering being observed on the day of showerhead installation and more disperse clustering in samples taken during early and mature biofilm development (**Supplementary Figure S5**). The membership in these samples also changes as showerhead age increases, which further suggests that biofilm formation in virgin plumbing fixtures impacts the composition of the microbiome in both water and aerosol samples (**Figure 6**). As the study progressed, samples significantly decreased in richness and diversity, but increased in evenness.

## 4 Discussion

There are few studies that focus on aerosolization from DW, and those that do use vastly different methods than those used in this study in both aerosol collection and quantification methodology. Speaking very broadly, other studies have found *L. pneumophila* (Collins et al., 2017) and *P. aeruginosa* (Chattopadhyay et al., 2017) present, but in low concentrations in shower water and even lower concentrations in shower aerosols when using culture-independent techniques, which yields comparable partitioning to that found in this study. The genus *Mycobacteria*, however, have been documented to make up large proportions of the DW microbial community (Haig et al., 2018; Dowdell et al., 2019), and its presence as a major community member in the sequencing and absolute quantification results is in agreement despite being detected in lower quantities than other studies (Shen et al., 2022). Interestingly, observations of NTM in aerosols in this study did not conform to the consensus within literature (Falkinham, 1996) that the genus is easily aerosolizable due to their hydrophobic cell membrane. This discrepancy could be attributed to the lack of size exclusion during aerosol collection in previous studies (i.e., collecting total NTM bioaerosols instead of respirable NTM bioaerosols), in addition other studies have collected samples from established plumbing sources of unknown ages, so it is conceivable that NTM aerosolizes better after more than 84 days. Regardless of showerhead type at the point of installation, respirable DWPIs that may be inhaled over the course of an average shower were found to be lower in abundance in this study (**Figure 2**) than in previous studies (Estrada-Perez et al., 2018; Shen et al., 2022). However, due to methodology differences in sampling time (60 minutes (Shen et al., 2022) compared to 20 minutes here), aerosol fraction collected (all sizes (Shen et al., 2022) compared to < 10 µm in this study) and aerosol collection instruments used it is not possible to compare these studies as truly equivalent, with the methodology of these other studies leading to higher bioaerosol counts inherently. There are many factors that contribute to showerhead aerosol generation such as number of water jets, orientation of jets on the showerhead, flow rate of shower water, and spray pattern (Zhou et al., 2019), all of which were controlled in this study but may differ in other studies.

### 4.1 Antimicrobial showerheads may not function as a microbial inhibitor, but microbial selector

The disparity between the marketing claims of these showerheads and their performance in a full-scale system could be due to a variety of factors, but likely are either due to material/antimicrobial agent or application issues. The lack of significant chlorine removal from the filter-based showerhead could be attributed to poor filter performance. Although the manufacturers do not report their testing methodology on chlorine removal, it could be possible that this significant reduction is seen in standardized laboratory solutions or in non-operational conditions. From an antimicrobial agent perspective, it is possible that the tested antimicrobial showerheads do not contain the agent, which would violate Title 15 of the United States Code Section 1125 stating general provisions against false descriptions 15 U.S.C. § 1125 (2020), however without extensive material testing which was outside of the scope of this study, this potential explanation cannot be further explored. What is more likely, however, to explain the lack of difference in microbial abundance could be due to too low of a concentration of the antimicrobial agent (silver and copper in the case of the silver-embedded and filter-based showerheads, respectively) to effectively inactivate microbes during a short exposure/contact time within a showerhead and shower hose (seconds to minutes). According to the manufacturer of the silver-embedded head, the active agent is tested in accordance with ISO 22196, which involves assessing the reduction of *Staphylococcus aureus* and *E. coli* on the antimicrobial material in nutrient abundant conditions after 24 h of incubation (In2itive, 2011). These conditions are vastly different from the shower water and showerhead environments and the organisms used are not commonly found in DW and don’t represent the DWPI’s claimed to be removed. Additionally, it is possible that the antimicrobial showerheads may be effective during a longer time of operation (after 84 days) or when DWPI concentrations are significantly higher. The former explanation is unlikely, since the manufacturer suggests replacing the silver-embedded showerhead after 60 days of use. It should however, be stressed that none of the showerheads tested in this study have an National Science Foundation (NSF) or the American National Standards Institute (ANSI) certifications for specific contaminant removal, so the manufacturers claims have not been tested to the voluntary standards used in the U.S (NSF Standards for water treatment systems, 2023). Furthermore, this study used molecular methods which detect both live and dead DWPIs, so it is possible differences may exist if methodologies that accounted for viability were solely used. Future work should consider the use of nucleic acid intercalating dyes or the evaluation of RNA to assess DWPI dynamics, however both methods have several challenges to ensure accuracy (Wang et al., 2019) The linear models generated from the absolute abundance data identified showerhead type to only explain a fraction of the variance in the models for *L. pneumophila* and NTM in the water phase, which further indicates that the showerhead material in this study was not a driving factor in DWPI abundance in either phase or partitioning.

Antimicrobial showerheads were expected to decrease the number of genera in both phases due to their marketed bactericidal properties, however the sequencing results illustrated that the silver-embedded and filter-based showerheads supported growth of microbial genera not recovered from the conventional ABS plastic showerhead. There is a large body of work that suggests that the type of material used in DW plumbing significantly alters the microbiome, but these studies have only been conducted on conventional piping materials (Cullom et al., 2020; Wang et al., 2014; Proctor et al., 2016; Proctor et al., 2017; Logan-Jackson et al., 2023). This trend of material influencing the microbiome is likely also occurring inside these antimicrobial showerheads and may explain the differences in community membership. For example, the highest number of taxa being found in the filter-based showerheads makes sense as it has been documented that filters can be colonized by microorganisms and support a large array of microbial growth on the unit process scale (Velten et al., 2011; LaPara et al., 2015), however understanding the material effects of the showerhead itself on microbial community must be further explored to determine if there are unintended consequences to using novel showerhead materials.

### 4.2 Plumbing age as a factor in DWPI load in water and aerosols

In addition to showerhead type and sample type being known to impact the DW microbial community, the age of plumbing and associated fixtures has been shown to impact the microbiome (Proctor et al., 2016) more so than the showerhead type in this study, as seen in the linear models generated from the absolute quantification data. As the plumbing is used over time, the material in contact with the DW can be affected by the water chemistry and microbiome. Biofilm colonization is a very common process because of this, but little is known about DW biofilm kinetics and their effects on water quality and bioaerosol formation (Costerton et al., 1995; Morvay et al., 2011). The abundance trends observed over the course of the study in the water samples for each DWPI correlate with what little is known about DW biofilm formation dynamics: *L. pneumophila* and *P. aeruginosa* have different biofilm formation dynamics and thus may detach from the biofilm and enter the bulk water phase at varying and unpredictable points during the biofilm formation process (Falkinham et al., 2015b), (Tart and Wozniak, 2008). NTM is a known early colonizer of DW biofilms (Falkinham, 2018; Dowdell et al., 2019) and forms fairly consistent robust biofilms on common plumbing materials after as little as 7 days (Mullis and Falkinham, 2013), so it is possible that the observed increase in water samples came from sloughing of a mature biofilm after day 42. Although biofilm formation kinetics and characterization in DW systems is an emerging area of research, these changes in alpha diversity may be influenced by the sessile community within the hose and showerhead sloughing into the water as the biofilm matures (Costerton et al., 1995). Such structural convergence has been documented in previous studies in DW distribution systems after different treatment processes (Shaw et al., 2014), and in a shower hose material study which initially saw differing microbial densities across material types but a convergence over time (Proctor et al., 2016). The core genera such as *Acidovorax*, *Sphingomonas*, and *Stenotrophomonas* in the sequencing results also suggest that biofilm forming microorganisms are major members of the microbiome (**Figure 4B**) (Jia et al., 2015; Delafont et al., 2016; Gulati and Ghosh, 2017). Although DWPI risk cannot be assessed due to the lack of viability data, these results demonstrate differing DWPI potential risk dynamics as a function of time (and subsequent biofilm formation). These differences need to be explored further using both culture-based approaches and molecular methods targeting viable DWPIs as they suggest that quantitative microbial risk assessment for each DWPI should factor in the age of the showerhead.

Overall, antimicrobial showerheads did not have significantly different water chemistry or DWPI abundances in shower water and shower water associated aerosols over the duration of the study. Despite not changing the absolute abundances of DWPIs or total bacteria, showerhead type impacted the microbial community of the water and aerosols which may indicate that there are material effects beyond the marketed antimicrobial properties that may be impacting microbial growth or establishment, and that biofilm development is an integral part of the shower system. Aerosolization behavior of DWPIs was found to be the same across all showerhead types, however the proportion and time frame of maximum aerosolization varied for each DWPI studied. Although DWPI risk cannot be assessed due to the lack of viability testing, these findings suggest that future quantitative microbial risk assessment for DWPIs should consider the showerhead age. There are many experimental considerations for this work, most notably that samples were analyzed for DNA and not RNA, so viability was not considered in this study, in addition to lacking the extensive materials and product design testing of these showerheads to confirm their marketed properties in the laboratory environment. Future work should also include in-depth materials testing of showerhead and hose material to independently assess and verify their antimicrobial properties in the DW environment. Additionally, temporal characterization of microbiome and DWPI abundance within the water, biofilm and aerosol phases in full-scale model studies like this one are required beyond the 84 days of this study to determine if the observed dynamics change, especially for NTM. The inclusion of a human analog (i.e. mannequin) should also be considered for subsequent studies to assess the changes in aerosolized DWPI deposition and to relate the findings more closely to consumer exposure. Based on the results of this study, a consumer choosing between a conventional or antimicrobial showerhead may want to install a cost-effective conventional showerhead which achieves similar chemical and microbial quality to the more expensive antimicrobial alternatives.

## Data availability statement

The datasets presented in this study can be found in online repositories. The names of the repository/repositories and accession number(s) can be found in the article/**Supplementary Material**.

## Author contributions

SP: Data curation, Formal Analysis, Investigation, Methodology, Visualization, Writing – original draft, Writing – review & editing. S-JH: Conceptualization, Funding acquisition, Resources, Supervision, Writing – review & editing.
