# Supplementary material for: Assessing the impact of anti-microbial showerheads on the prevalence and abundance of opportunistic pathogens in shower water and shower water-associated aerosols
Source: Front Microbiomes. 2023 Nov 2;2:1292571. doi: 10.3389/frmbi.2023.1292571 (PMC12993570; doi:10.3389/frmbi.2023.1292571)
Supplement: Supplementary file 1 [file DataSheet_1.docx]

Supplementary Material

**Contents:**

Supplementary Table 1. Different physical and chemical water quality parameters measured in this study and corresponding analytical method used………………………………………………..…….2

Supplementary Table 2. Molecular **primers, thresholds, and assay sensitivity for ddPCR analysis…..3**

Supplementary Table 3. Average values of measured water chemistry parameters…………………..4

Supplementary Table 4. Average aerosol partitioning values of DWPIs and total bacteria by head type……………………………………………………………………………………………………..7

Supplementary Procedure 1. Threshold determination for ddPCR quantification……………………8

Supplementary Figure 1. Schematic of full-scale shower laboratory set-up……………………...…..9

Supplementary Figure 2. Absolute gene copy concentrations separated by showerhead type over time…………………………………………………………………………………………………..10

Supplementary Figure 3. Absolute gene copy concentrations in shower water associated aerosols over time……………………………………………………………………………………………..11

Supplementary Figure 4**.** Absolute gene copy concentrations in shower water over time…………..12

Supplementary Figure 5. Non-metric Multidimensional Scaling plot by sample type (A.) and biofilm age bin of water samples (B.)………………………………………………….………..……………13

## Supplementary Tables

**Supplementary Table 1.** Different physical and chemical water quality parameters measured in this study and corresponding analytical method used.

| **Parameter** | **Units** | **Analytical Technique** | **LOD/LOQ** |
| --- | --- | --- | --- |
| Temperature | °C | Thermometer |  |
| pH |  | pH electrode |  |
| Free Chlorine | mg/L as Cl2 | DPD method | 0.01/0.02 |
| Total Chlorine | mg/L as Cl2 | DPD method | 0.01/0.02 |
| Ammonia | mg/L as NH3-N | Salicylate method | 0.01/0.01 |
| Orthophosphate | mg/L as PO4³¯ | Ascorbic acid method | 0.01/0.02 |
| Total Organic Carbon | mg/L | TOC analyzer | 0.01/0.01 |
| Dissolved Organic Carbon | mg/L | TOC analyzer (0.45 µm filtered) | 0.01/0.01 |
| Total Iron | mg/L | ICP-MS | 0.01/0.01 |
| Total Copper | mg/L | ICP-MS | 0.01/0.01 |
| Total Silver | mg/L | ICP-MS | 0.01/0.01 |
| Total Lead | mg/L | ICP-MS | 0.01/0.01 |
| Total Calcium | mg/L | ICP-MS | 0.01/0.01 |
| Total Magnesium | mg/L | ICP-MS | 0.01/0.01 |
| Dissolved Iron | mg/L | ICP-MS (0.45 µm filtered) | 0.01/0.01 |
| Dissolved Copper | mg/L | ICP-MS (0.45 µm filtered) | 0.01/0.01 |
| Dissolved Silver | mg/L | ICP-MS (0.45 µm filtered) | 0.01/0.01 |
| Dissolved Lead | mg/L | ICP-MS (0.45 µm filtered) | 0.01/0.01 |
| Dissolved Calcium | mg/L | ICP-MS (0.45 µm filtered) | 0.01/0.01 |
| Dissolved Magnesium | mg/L | ICP-MS (0.45 µm filtered) | 0.01/0.01 |

**Supplementary Table 2.** Molecular **primers, thresholds, and assay sensitivity for ddPCR analysis.**

| **Target Species** | **Forward (5'-3')** | **Reverse (5'-3')** | **Approx. Amplicon Size (bp)** | **Ref** | **Limit of Detection (copies/20 µL)** | **Limit of Quantification (copies/20 µL)** | **Threshold for water samples** | **Threshold for aerosol samples** |
| --- | --- | --- | --- | --- | --- | --- | --- | --- |
| *Legionella pneumophila* | *LpneuF:* | *LpneuR:* | 150 | 1 | 6.08 | 6.08 | 8800 | 6500 |
| *Lmip* gene | CCGATGCCACATCATAGC | CCAATTGAGCGCCACTCATAG |  |  |  |  |  |  |
| *Pseudomonas aeruginosa* | Ps-F: | Ps-R: | 117 | 2 | 7.3 | 7.3 | 4500 | 12530 |
| *Orpl* gene | CGAGTACAACATGGCTCTGG | ACCGGACGCTCTTTACCATA |  |  |  |  |  |  |
| *Nontuberculous mycobacteria* | FatpE: | RatpE: | 164 | 3 | 5.6 | 5.6 | 10600 | 9650 |
| *atpE* gene | CGGYGCCGGTATCGGYGA | CGAAGACGAACARSGCCAT |  |  |  |  |  |  |
| *Total bacteria* | Eub338: | Eub518: | 200 | 4 | 5.3 | 53 | 12900 | 11100 |
| *16s rRNA* gene | ACTCCTACGGGAGGCAG | ATTACCGCGGCTGCTGG |  |  |  |  |  |  |
| **Thermocycling Conditions**: 95 °C for 5 min, [95 °C for 0.5 min, 57 °C for 1 min, 72 °C for 1 min] x 45, 4 °C for 5 min, 90 °C for 5 min | | | | | | | | |
| **References: 1.** Wullings, B. A. et al. 2011. Appl Environ Microbiol 77 (2), 634-641 **2.** Feizabadi MM et al. 2010. Infect Genet Evol 10: 1247-1251 **3**. Radomski, N., et al., 2013. BMC Microbiol, 13(1), 277 **4.** Fierer, N., et al., 2005. App, Env, Microbiol, 71(7), 4117-4120 | | | | | | | | |

**Supplementary Table 3**. Average values of measured water chemistry parameters.

|  | | | | | | | | **Total** | | | | | | | **Dissolved** | | | | | | |
| --- | --- | --- | --- | --- | --- | --- | --- | --- | --- | --- | --- | --- | --- | --- | --- | --- | --- | --- | --- | --- | --- |
| **Age** | **Type** | **pH** | **Temp (°C)** | **Free Cl** | **Total Cl** | **Ammonia** | **Ortho-PO_4_^3-^** | **Org C** | **Fe** | **Cu** | **Ag** | **Pb** | **Mg** | **Ca** | **Org C** | **Fe** | **Cu** | **Ag** | **Pb** | **Mg** | **Ca** |
| **0** | **ABS Plastic** | 8.6±0.07 | 36.2±0.4 | 0.01±0 | 0.01±0 | 0.1±0.06 | 1.3±0.2 | 1.5±0.05 | 0.1±0.02 | 0.03±1x10^-3^ | <LOD | <LOD | 12.1±0.3 | 33.9±0.06 | 1.4±0.1 | 0.1±0.03 | 0.04±0.02 | <LOD | <LOD | 12.1±0.5 | 34.2±0.6 |
|  | **Silver-embedded** | 8.l±0.7 | 37.2±0.7 | 0.01±0 | 0.0±0 | 0.04±0.02 | 1.7±0.04 | 1.5±0.3 | 0.1±0.03 | 0.04±4x10^-3^ | <LOD | <LOD | 9.2±0.6 | 32.5± 2.1 | 0.9±0.03 | 0.2±0.1 | 0.03±7.7x10^-3^ | <LOD | <LOD | 9.7±0.3 | 33.4±0.3 |
|  | **Filter-based** | 8.l±0.05 | 36.9±0.8 | 0.05±0.1 | 0.02±0.02 | 0.02±0 | l.5±0.2 | l.4±0.2 | 0.2±0.03 | 0.07±0.02 | <LOD | <LOD | 9.1±0.3 | 34.1±1.1 | 1.1±0.1 | 0.2±0.01 | 0.05±3.3x10^-3^ | <LOD | <LOD | 9.2±0.2 | 34.3±0.8 |
| **14** | **ABS Plastic** | 8.l±0.05 | 36.8± 1.1 | 0.2±0.06 | 0.09±0.02 | 0.03±0.01 | l.2±0.1 | 1.2±0.2 | 0.3±0.07 | 0.04±1x10^-3^ | <LOD | <LOD | 9.4±0.3 | 34.3±1.6 | 0.2±0.2 | 0.2±0.03 | 0.04±2.5x10^-3^ | <LOD | <LOD | 9.7±0.2 | 34.5±0.5 |
|  | **Silver-embedded** | 8.0± 5x10^-3^ | 37.8±0.3 | 0.2±0.07 | 0.1±0.1 | 0.02±0.02 | 1.1±0.2 | 1.3±0.06 | 0.2±0.1 | 0.04±0.02 | <LOD | <LOD | 9.5±0.3 | 35.7±0.7 | 0.5±0.8 | 0.3±0.02 | 0.09±0.09 | <LOD | <LOD | 9.4±0.1 | 34.9±0.6 |
|  | **Filter-based** | 8.0±0.02 | 36.8±1.5 | 0.04±0.04 | 0.09±0.03 | 0.04±0.01 | 1.1±0.5 | 1.3±0.2 | 0.3±0.01 | 0.04±4.5x10^-3^ | <LOD | <LOD | 9.4±0.2 | 36.5±0.6 | 1.6±0.4 | 0.7±0.6 | 0.1±0.09 | <LOD | <LOD | 22.6±22.5 | 90.7±93.9 |
| **28** | **ABS Plastic** | 8.5±0.02 | 36.9±0.8 | 0.02±0.03 | 0.07±0.02 | 0.01±0.01 | 1.2±0.2 | 0.9±0.2 | 0.4±0.03 | 0.03±3.4x10^-3^ | <LOD | <LOD | 10.5±0.1 | 36.2±0.5 | 2.0±0.2 | 0.4±0.05 | 0.03±4.4x10^-3^ | <LOD | <LOD | 10.7±0.2 | 36.1±0.1 |
|  | **Silver-embedded** | 8.l±0.04 | 38.4± 1.4 | 0.12±0.08 | 0.08±0.01 | 0±0 | 1.2±0.2 | 1.0±0.1 | 0.5±0.02 | 0.05±4.9x10^-3^ | <LOD | <LOD | 9.1±0.07 | 36.4±1.0 | 0.5±0.2 | 0.5±8.8x10^-3^ | 0.04±7.3x10^-3^ | <LOD | <LOD | 9.1±0.1 | 36.9±0.8 |
|  | **Filter-based** | 8.0±0.05 | 37.6±1.5 | 0.1±0.08 | 0.1±0.02 | 0.02±0.01 | l.2±0.1 | 1.1±0.2 | 0.5±0 | 0.05±7.3x10^-3^ | 0.02±0.01 | <LOD | 8.8±0.06 | 36.6±0.3 | 0.8±0.1 | 0.5±0.02 | 0.05±4.5x10^-3^ | <LOD | <LOD | 8.8±0.1 | 37.3±1.1 |
| **42** | **ABS Plastic** | 7.9±0.06 | 37.8±0.7 | 0.1±0.05 | 0.1±0.05 | 0.01±0.01 | 0.8±0.6 | 0.4±0.2 | 0.2±0.01 | 0.1±0.2 | <LOD | <LOD | 7.5±0.09 | 41.3±0.6 | 0.2±0.3 | 0.2±0.06 | 0.03±5.9x10^-3^ | <LOD | <LOD | 7.4±0.3 | 43.6±5.8 |
|  | **Silver-embedded** | 7.9±0.04 | 37.8±1.2 | 0.1±0.02 | 0.2±0.08 | 0.01±0.01 | 0.6±0.6 | 0.4±0.2 | 0.2±0.03 | 0.04±1.1x10^-3^ | <LOD | <LOD | 7.6±0.05 | 42.7±1.6 | 0.5±0.7 | 0.2±0.02 | 0.05±0.0I | <LOD | <LOD | 7.5±0.2 | 43±2.0 |
|  | **Filter-based** | 7.9±0.04 | 37.1±1.1 | 0.1±0.05 | 0.04±0.01 | 0.02±0.02 | 1.2±0.07 | 0.4±0.05 | 0.2±0.01 | 0.05±4.8x10^-3^ | <LOD | <LOD | 7.6±0.2 | 45.1±0.6 | 0.4±0.5 | 0.2±0.03 | 0.05±5.4x10^-3^ | <LOD | <LOD | 7.9±0.8 | 47.5±5.3 |
| **56** | **ABS Plastic** | 7.9±0.03 | 36.8±0.6 | 0.06±0.03 | 0.1±0.04 | 0.1±0.05 | 1.2±0.06 | 2.6±0.2 | 0.2±0.03 | 0.05±4.7x10^-3^ | <LOD | <LOD | 8.3±0.1 | 54.5±1.2 | 2.7±0.02 | 0.2±0.02 | 0.04±5.5x10^-3^ | <LOD | <LOD | 8.0±0.2 | 52.8±1.7 |
|  | **Silver-embedded** | 7.8±0.02 | 38.3± 1.1 | 0.05±0.03 | 0.06±0.03 | 0.02±0.01 | 1.1±0.03 | 2.4±0.1 | 0.2±0.02 | 0.05±4.7x10^-3^ | <LOD | <LOD | 8.2±0.07 | 54.5±0.8 | 2.5±0.2 | 0.2±0.02 | 0.04±5.5x10^-3^ | <LOD | <LOD | 8.1±0.09 | 56.2±1.2 |
|  | **Filter-based** | 7.8±0.03 | 37.2±1.4 | 0.02±0.03 | 0.04±0.02 | 0.03±0.02 | 1.1±0.3 | 2.6±0.2 | 0.2±0.02 | 0.05±2.6x10^-3^ | <LOD | <LOD | 8.3±0.2 | S6.6±1.0 | 3.0±0.4 | 0.2±0.03 | 0.05±0.01 | <LOD | <LOD | 9.0±1.3 | 61.0±8.8 |
| **70** | **ABS Plastic** | 7.9±0.05 | 37.0±1.6 | 0.07±0.09 | 0.06±0.03 | 0.01±0.01 | 1.2±0.04 | 1.5±0.3 | 0.2±0.02 | 0.05±4.8x10^-3^ | <LOD | <LOD | 7.5±0.09 | 50.4±0.3 | 1.4±0.1 | 0.2±0.02 | 0.04±2.0x10^-3^ | <LOD | <LOD | 7.5±0.2 | 51.9±1.4 |
|  | **Silver-embedded** | 7.8±0.03 | 38.0±1.0 | 0.05±0.01 | 0.07±0.01 | 0±0 | 0.9±0.8 | 1.2±0.1 | 0.2±0.02 | 0.06±9.8x10^-3^ | <LOD | <LOD | 7.1±0.06 | 51.1±0.7 | 2.0±0.3 | 0.2±0.03 | 0.05±7.2x10^-3^ | <LOD | <LOD | 7.0±0.05 | S0.4±0.4 |
|  | **Filter-based** | 7.S±0.03 | 37.5±1.2 | 7x10^-3^±0.01 | 0.05±0.03 | 0.02±0.03 | 0.7±0.6 | 1.1±0.05 | 0.2±0.02 | 0.06±4.5x10^-3^ | <LOD | <LOD | 7.0±0.2 | 50.0±0.5 | 2.1±0.3 | 0.2±0.06 | 0.06±5.5x10^-3^ | <LOD | <LOD | 6.9±0.09 | 51.6±0.6 |
| **84** | **ABS Plastic** | 7.8±0.04 | 37.1±1.1 | 0.05±0.04 | 0.05±0.01 | 0.07±0.06 | 1.2±0.3 | 0.4±0.7 | 0.1±0.03 | 0.04±0.01 | <LOD | <LOD | 6.0±0.1 | 43.5±0.6 | 0.4±0.7 | 0.1±0.03 | 0.03±4.1x10^-3^ | <LOD | <LOD | 0.6±0.02 | 0.6±1.2 |
|  | **Silver-embedded** | 7.8±0.08 | 38.0±1.1 | 0.02±0.01 | 0.03±0.03 | 0.04±0.03 | l.2±0.5 | 0.5±0.8 | 0.2±0.1 | 0.7±0.8 | <LOD | 1.2x10^-2^±3x10^-3^ | 6.6±0.7 | 45.7±1.4 | 1.4±0.1 | 0.2±0.02 | 0.2±0.1 | <LOD | 0.01±1.3x10^-3^ | 0.6±0.07 | 44.7±0.2 |
|  | **Filter-based** | 7.7±0.03 | 37.5±0.7 | 0.01±0 | 0.04±0.02 | 0.04±0.02 | 1.1±0.5 | 0.7±0.7 | 0.2±0.08 | 0.2±1.2 | <LOD | <LOD | 6.2±0.3 | 46.2±1.8 | 0.8±0.7 | 0.1±0.04 | 0.08±0.02 | <LOD | 0.01±3.9x10^-3^ | 0.6±0.02 | 44.9±1.4 |

**Supplementary Table 4**: Average aerosol partitioning values of DWPIs and total bacteria by head type

| **Target** | **Average aerosolization partitioning percentage (%) ± standard deviation** | | | **Significance of showerhead type (ANOVA *p-*value)** |
| --- | --- | --- | --- | --- |
|  | **ABS Plastic** | **Silver-embedded** | **Filter-based** |  |
| *L. pneumophila* | 1.06 ± 2.1 | 3.27 ± 5.8 | 1.71 ± 3.0 | 0.3 |
| *P. aeruginosa* | 5.33x10^-5^ ± 1.34x10^-4^ | 1.07x10^-4^ ± 2.35x10^-4^ | 2.16x10^-5^ ± 5.85x10^-5^ | 0.2 |
| Nontuberculous mycobacteria | 1.02x10^-6^ ± 3.22x10^-6^ | 1.14x10^-6^ ± 3.17x10^-6^ | 2.8x10^-7^ ± 8.34x10^-7^ | 0.2 |
| Total bacteria | 1.74x10^-5^ ± 2.67x10^-5^ | 3.28x10^-5^ ± 4.90x10^-5^ | 1.76x10^-5^ ± 2.13x10^-5^ | 0.5 |

**Supplementary Procedures**

**Supplementary Procedure 1.** Threshold determination for ddPCR quantification

Thresholding was achieved by taking a sample that had already been run on the instrument, and spiking a known amount of gblock into the matrix for each primer type used in this study. Spiked samples were run using the same method described in the manuscript, and the amplitude plots for the spiked and unspiked sample were compared. Thresholds were tried between the positive and negative bands: each concentration output for potential threshold was back calculated, and the concentration of the unspiked sample was subtracted from the spiked sample concentration. This process was iterated until a threshold was chosen that yielded a spiked sample concentration that matched the initial gblock concentration initially put into the sample after the subtraction of the concentration of the unspiked sample.

**Supplementary Figures**


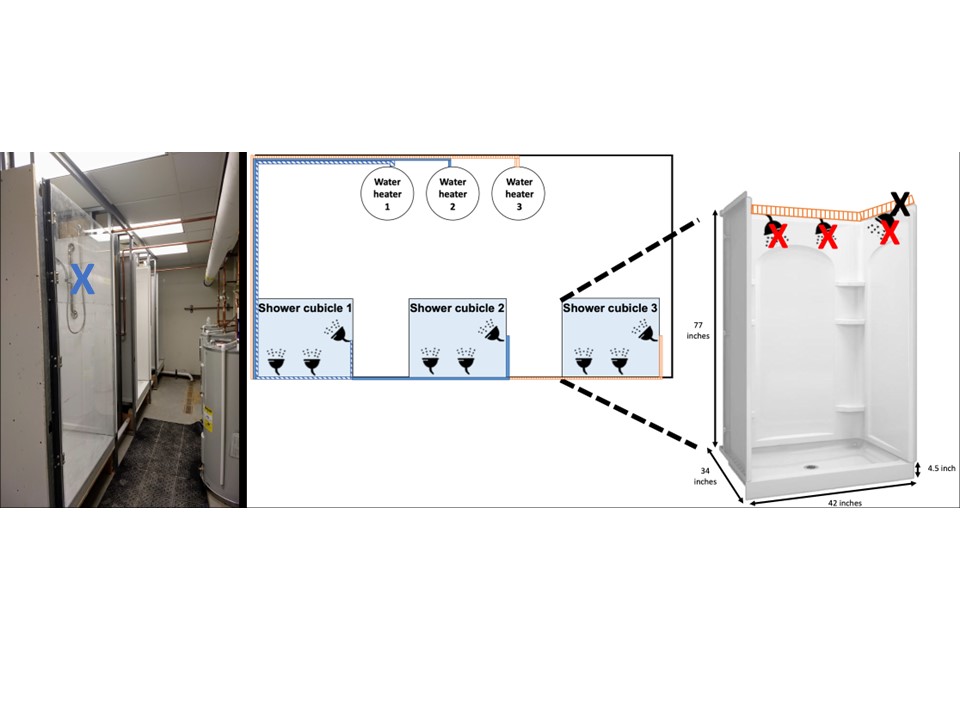


**Supplementary Figure 1**. Schematic of full-scale shower laboratory set-up. The blue X indicates the aerosol sampling port in the Plexiglass door, the black X indicates where the copper pipe from the hot water heater connects to the shower stall, and the red X’s correspond to each showerhead position.


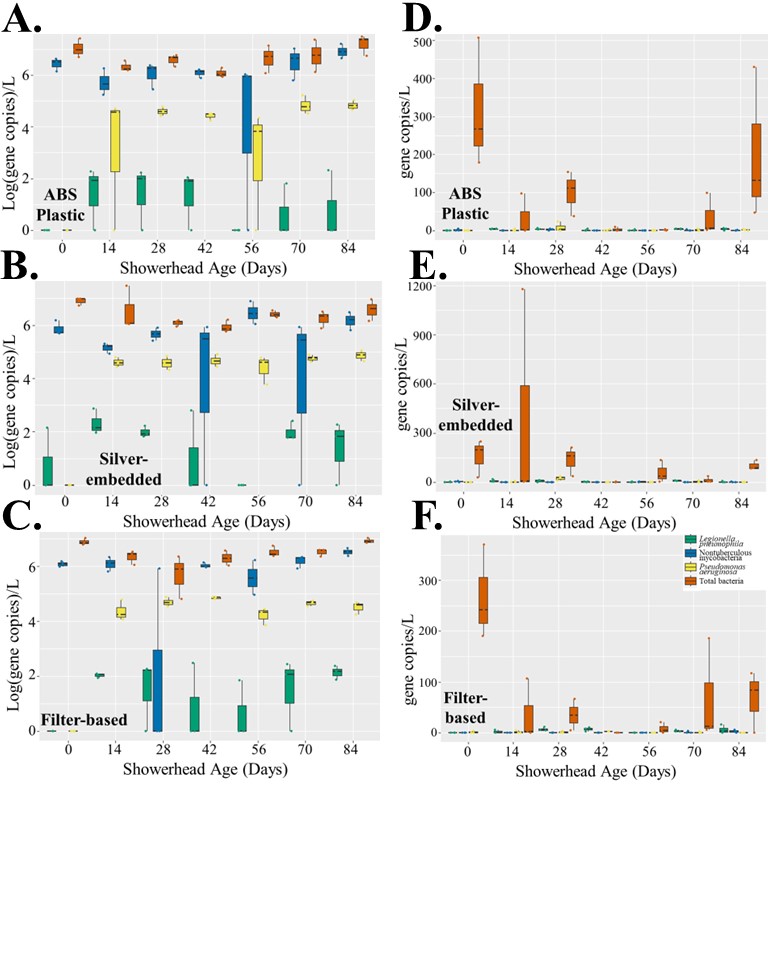


**Supplementary Figure 2.** Absolute gene copy concentrations separated by showerhead type over time. **A.** water samples from ABS Plastic showerheads*,* **B.** water samples from silver-embedded showerheads*,* **C.** water samples from filter-based showerheads, **D.** aerosol samples from ABS Plastic showerheads, E. aerosol samples from silver-embedded showerheads, F. and aerosol samples from filter-based showerheads were sampled over 14 weeks of operation for *Legionella pneumophila* (green), *Pseudomonas aeruginosa* (yellow), nontuberculous mycobacteria (blue), and total bacteria (orange). Each showerhead type shows all the data collected from three experimental showerhead replicates for that specific time point.

**
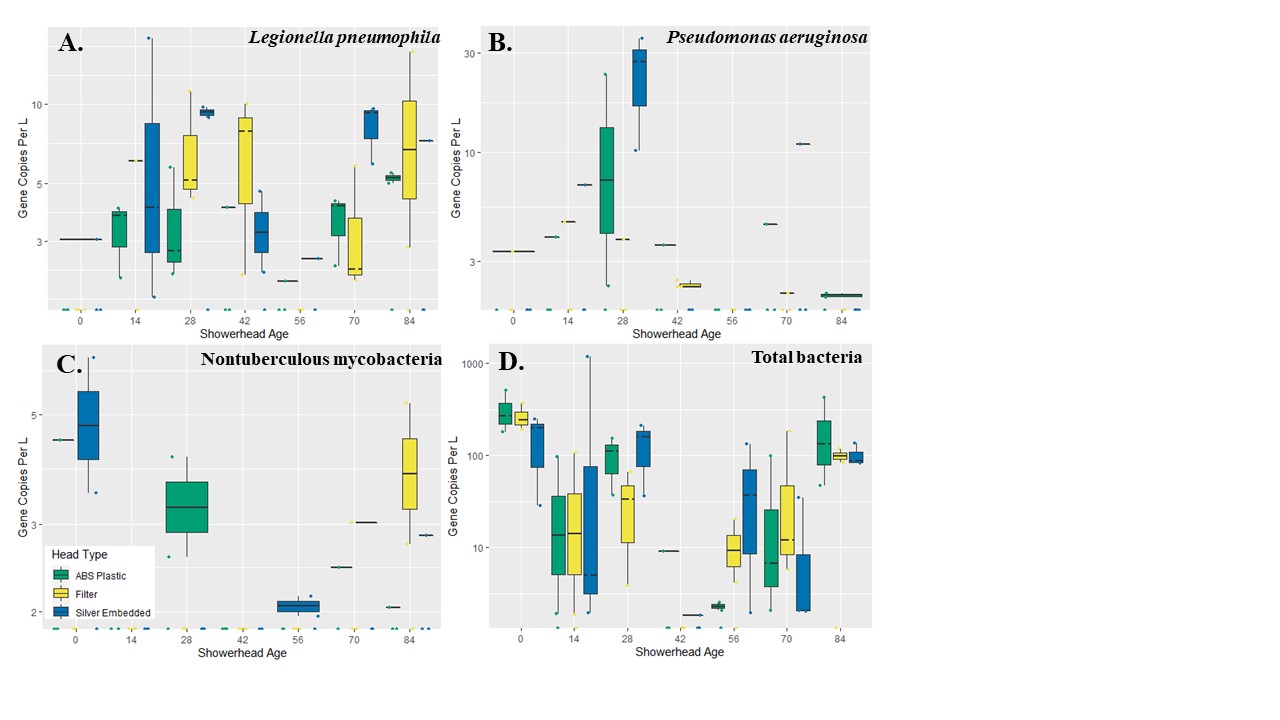
**

**Supplementary Figure 3.** Absolute gene copy concentrations in shower water associated aerosols over time. **A.** *Legionella pneumophila,* **B.** *Pseudomonas aeruginosa,* **C.** nontuberculous mycobacteria, and **D.** total bacteria observed across 14 weeks of operation in ABS plastic (green), filter-based antimicrobial (yellow), and silver-embedded (blue) showerheads in shower water associated aerosols. Each showerhead type shows all the data collected from three experimental showerhead replicates for that specific time point.

**
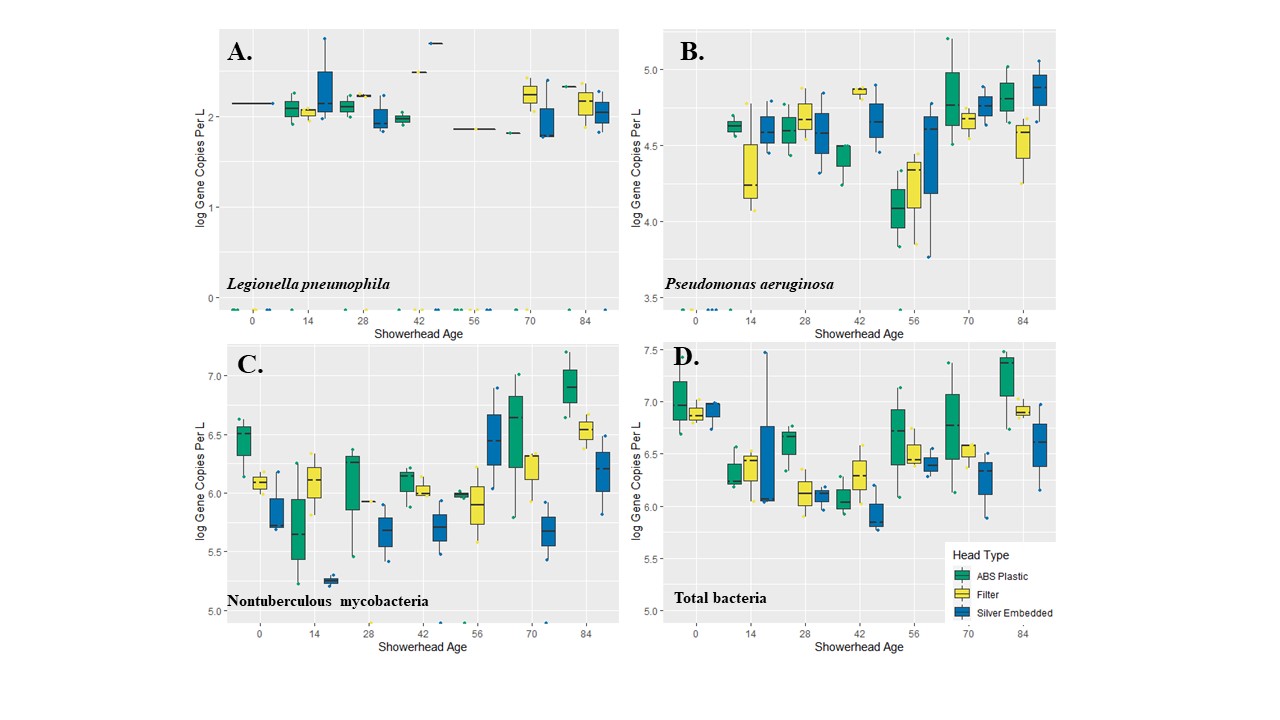
**

**Supplementary Figure 4.** Absolute gene copy concentrations in shower water over time. **A.** *Legionella pneumophila,* **B.** *Pseudomonas aeruginosa,* **C.** nontuberculous mycobacteria, and **D.** total bacteria observed across 14 weeks of operation in ABS plastic (green), filter-based antimicrobial (yellow), and silver-embedded (blue) showerheads in shower water. Each showerhead type shows all the data collected from three experimental showerhead replicates for that specific time point.


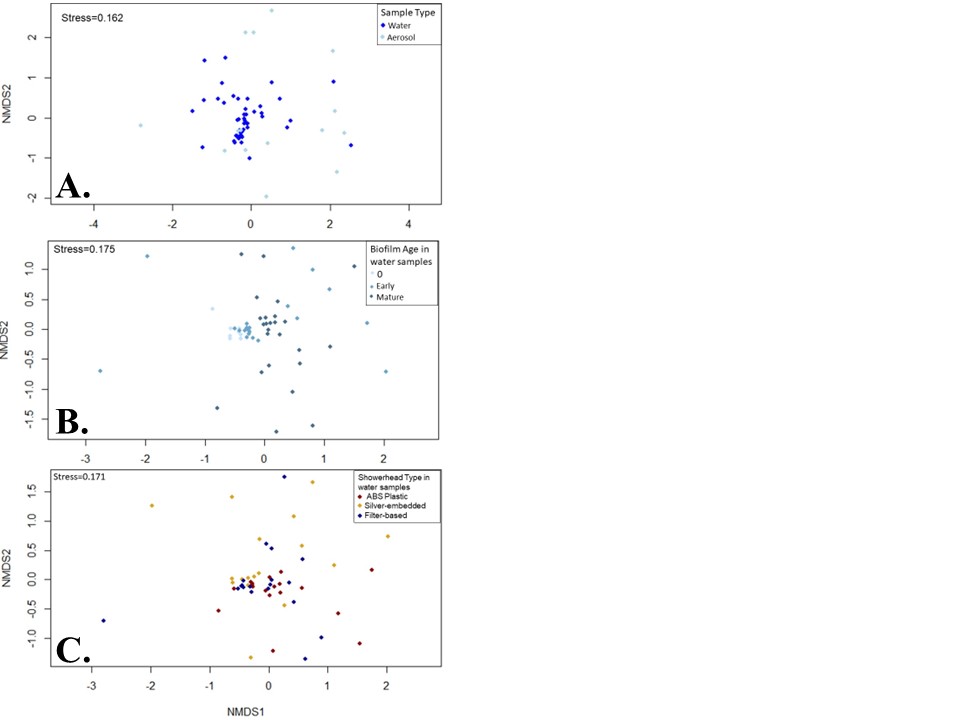


**Supplementary Figure 5.** Non-metric Multidimensional Scaling plot by A. sample type, B. biofilm age bin of water samples, and **C.** showerhead type of water samples.
